# Stepping closer to treating Alzheimer’s disease patients with BACE1 inhibitor drugs

**DOI:** 10.1186/s40035-016-0061-5

**Published:** 2016-07-14

**Authors:** Riqiang Yan

**Affiliations:** Department of Neurosciences, Lerner Research Institute, Cleveland Clinic Foundation, 9500 Euclid Avenue/NC30, Cleveland, OH 44195 USA

**Keywords:** Alzheimer’s disease, Amyloid plaques, Amyloid precursor protein, Secretase, BACE1, Aspartyl protease, Drug discovery, Clinical trials, Amyloid deposition, β-amyloid peptide, Fragment based drug discovery, Verubecestat

## Abstract

Alzheimer’s disease (AD) is the most common age-dependent neurodegenerative disease which impairs cognitive function and gradually causes patients to be unable to lead normal daily lives. While the etiology of AD remains an enigma, excessive accumulation of β-amyloid peptide (Aβ) is widely believed to induce pathological changes and cause dementia in brains of AD patients. BACE1 was discovered to initiate the cleavage of amyloid precursor protein (APP) at the β-secretase site. Only after this cleavage does γ-secretase further cleave the BACE1-cleaved C-terminal APP fragment to release Aβ. Hence, blocking BACE1 proteolytic activity will suppress Aβ generation. Due to the linkage of Aβ to the potential cause of AD, extensive discovery and development efforts have been directed towards potent BACE1 inhibitors for AD therapy. With the recent breakthrough in developing brain-penetrable BACE1 inhibitors, targeting amyloid deposition-mediated pathology for AD therapy has now become more practical. This review will summarize various strategies that have successfully led to the discovery of BACE1 drugs, such as MK8931, AZD-3293, JNJ-54861911, E2609 and CNP520. These drugs are currently in clinical trials and their updated states will be discussed. With the promise of reducing Aβ generation and deposition with no alarming safety concerns, the amyloid cascade hypothesis in AD therapy may finally become validated.

## Background

Alzheimer’s disease (AD) is the most common neurodegenerative disorder and is the third leading cause of death in the elderly behind heart disease and cancer. AD patients suffer from progressive cognitive decline in the form of dementia, with memory loss being the earliest sign. With life expectancy continually increasing, the number of AD cases is also growing rapidly, and therefore treating AD patients is becoming more urgent.

The typical neuropathological hallmarks of AD are the presence of extracellular amyloid plaques and intracellular neurofibrillary tangles (NFT), with pathology appearing initially in the hippocampus and then extending to the cortical grey matter [5, 64, 69, 83]. Amyloid plaques, also called senile plaques, result from the deposition of aggregated Aβ peptides, which can be naturally produced via sequential cleavages of amyloid precursor protein (APP) by the β- and γ-secretase [12, 28, 72]. A membrane-bound aspartyl protease called BACE1 was discovered as the β-secretase [41, 54, 71, 88, 92], while the γ-secretase is a complex consisting of four transmembrane proteins: presenilin-1 or −2, nicastrin, Aph1, and Pen2 [11, 20, 52, 53, 100]. Formation of NTF is due to paired helical filaments of the microtubule-binding protein tau in hyperphosporylated form [26, 47, 48]. Aβ, mainly Aβ_42_, appears to induce the formation of NFT, as implicated by mouse genetic studies [25, 39, 51, 62].

Over the past two decades, extensive knowledge concerning the molecular mechanisms underlying the formation of amyloid plaques and NFTs has been gained. However, the cause of AD still remains largely under debate. Studies from human genetics and mouse models indicate that abnormal production or accumulation of Aβ, especially the less soluble form of Aβ (42 or 43 amino acid long Aβ), appears to induce a cascade of synaptic dysfunctions commonly seen in AD patients [29, 50, 68, 85, 93]. Hence, targeting Aβ generation or clearance has been the major focus of research into AD therapy [24, 27, 59, 60]. Since BACE1 acts on the first and rate-limiting step in Aβ generation and BACE1 deficiency almost abolishes Aβ generation in mouse models, inhibition of BACE1 has received perhaps the most intensive efforts in academia and in industry. With the recent breakthrough in the development of brain-penetrable BACE1 inhibitors, several have advanced to clinical trials. In this review, we will update the progress of these BACE1 inhibitors in human studies.

## Discovery of BACE1 inhibitors

For effective and specific inhibition of BACE1, knowledge regarding the structure and function of BACE1 is essential. BACE1 belongs to the family of aspartic proteases, which typically have a bilobal structure, with each of its two active aspartate motifs [either DTG or DSG] located in a separate lobe; mutation of either aspartate residue renders an aspartic protease inactive [79, 82]. Among aspartic proteases, only BACE1 and BACE2 are localized on the cell membrane via the type I transmembrane domain. Although BACE1 and BACE2 share 59 % homology, they exhibit different enzymatic specificity. BACE1 mainly cleaves APP at the N-terminus of the Aβ domain (the β-secretase site) as well as the β’-site (between E10 and V11), while BACE2 preferentially cleaves APP within the Aβ region between F19 and F20 or between F20 and A21 [18, 73, 77, 94]. BACE1 deficiency almost abolishes the production of Aβ in mouse models, while mice deficient in BACE2 show no significant difference in Aβ generation [13]. Hence, BACE1 is viewed as the sole enzyme for initiating Aβ generation.

However, the crystal structure of BACE1, first solved by the Jordan Tang laboratory, indicates that the proteolytic site of BACE1 is largely similar to most human aspartic proteases, although it is more open and less hydrophobic [33]. These findings suggest potential cross-inhibition of BACE1 inhibitors, with other crucial aspartic proteases that are important to normal human functions, will need to be considered. Among these, cathepsin D and E as well as BACE2 have received the most attention. Mice deficient in cathepsin D manifest seizures and become blind near the terminal stage (postnatal day 26) ([46]. Cathepsin D knockout mice also develop granular osmiophilic deposits and abnormal autophagosomes in neonatal neurons [46] as well as persistent neuropathy related to dysfunctional lysosomes [70]. Mice deficient in cathepsin E show disrupted immune function due to systemic accumulation of IL-18 and IL-1β [84] as well as two major lysosomal membrane sialoglycoproteins, LAMP-1 and LAMP-2 [96]. The mechanism for these defects is mainly through reduced turnover rates. On the other hand, mice with BACE2 deficiency have been initially reported to be fertile and healthy in general [13], suggesting less of a concern for cross-inhibition. More encouragingly, BACE2 has been shown to regulate functions of pancreatic β-cells by shedding the proliferative plasma membrane protein Tmem27 [8, 16]. In rat pancreatic β- cells engineered to overexpress islet amyloid polypeptide, BACE2 silencing can significantly increase glucose-stimulated insulin secretion [2], suggesting a possible beneficial effect for BACE2 inhibition for type 2 diabetic patients. Nevertheless, BACE2 has also been shown to cleave PMEL17 by inhibiting melanosome maturation in melanocytes [67, 86]. Hence, for cross-inhibition considerations, recombinant cathepsin D, E and BACE2 should at least be included in in vitro enzymatic assay experiments to validate specific effects of BACE1 inhibitors.

Discovery of BACE1 inhibitors must necessarily include establishing the physiological functions of BACE1 in human. The generation of BACE1 knockout mice has been tremendously useful for understanding the role of BACE1 in vivo. Initial reports of BACE1-null mice suggested that BACE1 deficiency in mice posed no major concern, as BACE1-null mice appeared to be healthy [6, 56, 66]. Additional studies with BACE1-null mice, generated in different laboratories and facilities, have revealed indispensable roles of BACE1 in vivo such as the control of myelination [36, 38, 91], neuronal migration [3, 31, 49, 101], epileptic seizure [32, 40], neurogenesis and astrogenesis [37], formation of muscle spindle [9], etc. The development of these phenotypes is due to the cleavage of additional BACE1 substrates as discussed in detailed in recent reviews [4, 87, 89, 93, 95]. This knowledge is crucial for monitoring mechanism-based toxicity and for designing clinical trials.

## Early stages of BACE1 inhibitors

Immediately after the cloning of BACE1 in the late 1990s, the competition to produce active recombinant BACE1 enzymes for high-throughput screening (HTS) of BACE1 inhibitors in many pharmaceutical companies and academic labs became imminent and intense. The HTS method has the advantage of generating “small molecular hits” that often have high structural diversity and can easily be formulated for oral administration. Unfortunately, these intense efforts yielded few compounds with in vitro half-maximal inhibitory concentrations (IC_50_) in the nM range [23, 55]. This can be attributed to the large BACE1 catalytic pocket, which has a long substrate cleft and can accommodate a substrate with up to 11 residues [81]. Two noted winners in the HTS race were Wyeth and Roche. The small BACE1 inhibitory hits at Wyeth have a bicyclic amidine scaffold, while Roche discovered hits with a dihydrothiazine scaffold. Both companies further optimized their hits and developed more potent derivatives in the low μM range (38–40 μM), which showed reduced cerebrospinal fluid (CSF) or cortical Aβ in animal models [30, 57]. In spite of these initial successes, these BACE1 inhibitors were not advanced to clinical trials.

In addition to the HTS efforts, other rational designs of BACE1 inhibitors were simultaneously explored. The first successful substrate-based BACE1 inhibitor, developed by Elan Pharmaceuticals, was a P1 (S)-statine-substituted substrate analogue based on the sequence surrounding the β-secretase cleavage site (P10–P4′) with an IC_50_ of ~30 nM [71]. A more potent hydroxyethylene (HE) isotere-based transition-state analogue inhibitor, OM99-2, was developed by the Tang (Oklahoma Medical Research Foundation) and Ghosh (University of Illinois at Chicago/Purdue University) team shortly thereafter, with OM99-2 (Glu-Val-Asn-Leu-Ala-Ala-Glu-Phe) binding to its active site demonstrated by c-crystallization with BACE1, with a *K*_*i*_ near 1.0 nM [34]. Hydroxymethylcarbonyl (HMC) isostere-based transition-state analogs were also developed and optimized by Kiso’s group (Kyoto Pharmaceutical University), and eventually pentapeptidic BACE1 inhibitors showed comparable potency with the in vitro IC_50_ of KMI-420 being 8.2 nM and that of KMI-429 being 3.9 nM [44, 45]. Small-molecule hydroxyethylamine dipeptide isosteres also display high potency of BACE1 inhibition [74, 80]. Carbinamine-derived BACE1 inhibitors with the primary amine interacting with the catalytic Asp of BACE1 were also developed later and showed potency in the nM range [65]. Although these peptidomimetic BACE1 inhibitors are highly potent in vitro, their inherent poor drug properties (i.e., high total polar surface area with many rotatable bonds and numerous hydrogen bond donor acceptors) cause poor brain permeability. Peptidomimetic BACE1 inhibitors usually have a short half-life in vivo and low oral availability. This dilemma compels further exploration of later generations of smaller, non-peptidic BACE1 inhibitors in order to improve drug properties.

The noted successful example of this class is CTS-21166 from CoMentis [commented by [1]]. CoMentis revealed that CTS-21166 is a small transition-state analog inhibitor that has an IC_50_ in the range of 1.2–3.6 nM, has measurable brain penetration properties, exhibits over 100-fold selectivity over BACE2 and cathepsin D, shows metabolic stability, and can be orally administered [22]. When an AD mouse model was first tested by intraperitoneal (i.p.) injection (4 mg/kg over 6 weeks), CTS-21166 was shown to reduce brain Aβ levels by over 35 % and plaque load by 40 % [22]. These preclinical results were clearly encouraging and this compound became the first BACE1 inhibitor to pass a Phase I clinical trial in 2008. The data based on the CTS-21166 human Phase I trial in healthy young males indicated that this compound was safe at dose as high as 225 mg. When intravenously (i.v.) infused into AD patients, it caused a dose-dependent reduction of plasma Aβ levels with a nadir of approximately 80 % inhibition for the highest dosages at 3 h, and significant inhibition of plasma Aβ persisted beyond 72 h. The recovery of plasma Aβ to the pre-infusion level was nearly complete by 144 h after administration of the inhibitor. A second phase I trial on subjects receiving an oral liquid solution of 200 mg CTS-21166 showed similar efficacy [22]. Despite these initial encouraging results, CTS-21166 has not advanced further in clinical trials and the 6-year collaboration between Astellas Pharma and CoMentis for developing and commercializing CTD-21166 was terminated in 2014. In point of fact, transition analogs generally possess permeability glycoprotein efflux activity, which reduces central penetration/exposure. This class of compounds is therefore less favorable for central nervous system (CNS) drugs.

## Fragment-based discovery improves BACE1 inhibitor pharmacological properties

More successful BACE1 inhibitors have been developed through “fragment-based drug discovery” (FBDD) [see reviews by [10, 55, 75, 87]]. Compared to the traditional HTS approach, the FBDD approach takes advantage of biophysical techniques (NMR, X-ray co-crystallography, surface plasmon resonance (SPR), etc.) to screen libraries that consist of more diverse and smaller-sized compounds (fragments) for hits. Such hits can be further developed into potent leads with drug-like properties by medicinal chemists. A significantly higher hit ratio was obtained by using a FBDD approach, even though the binding affinity between the lower-molecular-weight fragments and the large active site of BACE1 is weaker. Among initial FBDD screening efforts, hits with amidine- or guanidine-containing heterocycles were commonly found to form a hydrogen-bonding network with the catalytic Asp residues of BACE1 [63]. Overall, the integration of various techniques in the fragment screening has made FBDD an increasingly popular method for designing potent small-molecule BACE1 inhibitors. Further optimized compounds with better CNS drug properties from initial hits are currently in clinical trials and will be summarized here.

### MK-8931 (Verubecestat)

**Fig. 1:**
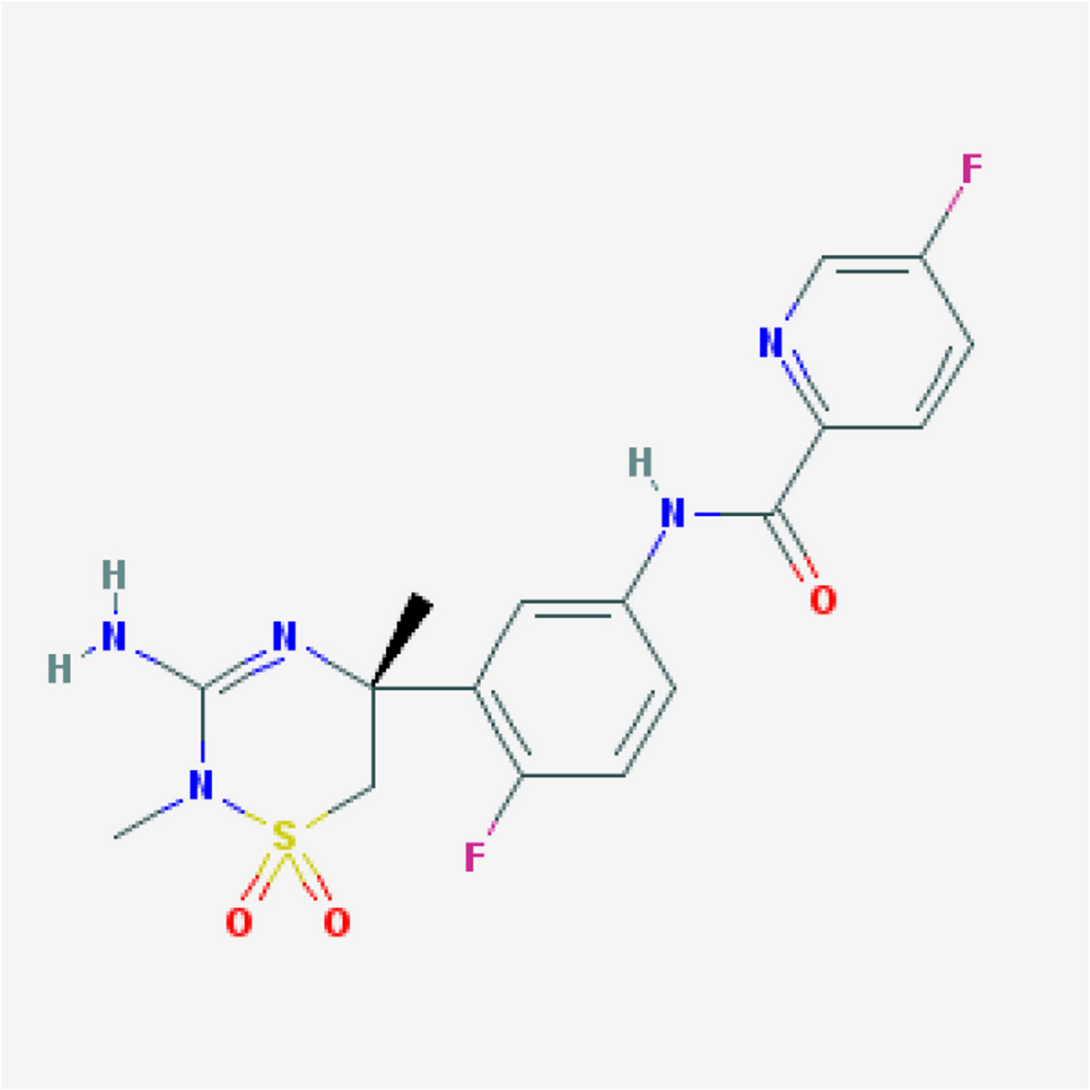
Chemical structure of compound MK-8931. MK-8931 is developed at Merck and also named as Verubecestat with a Chemical formula of C_17_ H_17_ F_2_ N_5_ O_3_ S. It’s structural name is N-[3-[(5R)-3-amino-5,6-dihydro-2,5-dimethyl-1,1-dioxido-2H-1,2,4-thiadiazin-5-yl]-4-fluorophenyl]-5-fluoro-2-pyridinecarboxamide. The molecular weight is 409.41

Merck’s BACE1 inhibitor MK-8931 was actually developed by the BACE1 team at Schering Plough (linked to SCH 900931 in the patent of US 20070287692 A1) through the FBDD method. In their first FBDD screening and quick optimization coupled with NMR and a functional assay, an isothiourea-containing hit with a Kd of 15 μM for BACE1 was first developed [90]. The H-bond of this hit interacts with the two catalytic aspartates within the active site of BACE1. Additional NMR-based searches for heterocyclic isothiourea isosteres, optimization of the 2-aminopyridine, as well as structure-based design of a cyclic acylguanidine led to BACE1 inhibitors with nanomolar Kd as well as better chemical stability and physicochemical properties [102]. Optimization of the pyrimidine substituent that binds in the S2′-S2″ pocket of BACE1 remediated time-dependent CYP3A4 inhibition of earlier analogues in this series and imparted high BACE1 affinity [58]. This compound, named MK-8931 or Verubecestat, has the formula of C_17_ H_17_ F_2_ N_5_ O_3_ S (see Fig. 1), and effectively reduces Aβ40 in cells with a Ki of 7.8 nM and an IC_50_ of 13 nM [76]. It also dramatically lowered CSF and cortex Aβ40 in both rats and cynomolgus monkeys following a single oral dose [58].

In 2010, MK-8931 was the first small molecular BACE1 inhibitor to enter a clinical phase I trial, with an initial evaluation in 88 people (40 for single doses of 100 or 450 mg in Belgium and 48 for multiple escalating doses of 12–150 mg in the USA). Another trial was targeted to people with renal insufficiency in order to assess efficient clearance of the drug. MK-8931 was presented as having excellent safety profiles with no immediately noticeable side effects. A single dose of MK-8931 reduces CSF Aβ concentrations in AD patients by as much as 92 %, while multiple low doses achieve a reduction of 50–80 % [19].

In Phase 1b randomized placebo-controlled trials, 32 individual with mild to moderate AD received 12 mg, 40 mg, and 60 mg of MK-8931 once daily for 7 days. Aβ40, Aβ42, and BACE1-cleaved secreted amyloid precursor protein β (sAPPβ) in CSF were collected and analyzed. Merck reported a dose-dependent and sustained reduction from baseline by 57, 79, and 84 % for CSF Aβ40 with these three dosages (http://www.mercknewsroom.com/press-release/alzheimers-disease/merck-presents-findings-phase-1b-study-investigational-bace-inhibit). Reductions in Aβ42 and sAPPβ were of a similar range. The results concerning safety, tolerability, pharmacokinetics, and pharmacodynamic profile support MK-8931 for testing at the next level.

In late 2012, Merck promptly launched combined Phase II/III clinical trials. This global EPOCH study would enroll 1960 individuals and is designed in two parts, Part I and Part II. Part I of the study is designed to assess the efficacy and safety of verubecestat (MK-8931) compared with placebo administered for 78 to 104 weeks for the treatment of AD patients with amnestic mild cognitive impairment, also known as prodromal AD. Participants randomized to receive one dose (12 or 40 mg verubecestat, once daily) will be compared with a placebo group for changes from baseline in the Clinical Dementia Rating scale-Sum of Boxes (CDR-SB) score at 78 or 104 weeks. Participants entering Part II will receive either 12 mg or 40 mg verubecestat, once daily for up to an additional 260 weeks. This combined trial will assess changes from baseline in cognition and function using several tests, but will also assess AD biomarkers, such as CSF total tau, hippocampal volume, and brain amyloid (Table 1). It is expected that Merck will report results from this study between 2018 and 2021.

**Table 1:** BACE1 inhibitors in clinical trials

Drug	MK-8931	JNJ-54861911	AZD-3293	E2609	CNP520
Sponsor	Merck	Janssen, Shionogi Pharma	AstraZeneca, Eli Lilly	Eisai, Biogen	Novartis, Amgen
Trial # (Phase)	NCT01739348 (II/III)	NCT02406027 (II)	NCT02245737 (II/III)	NCT02322021 (II)	NCT02576639 (II)
	NCT01953601 (II/III)	NCT02406027 (II)			NCT02565511 (II/III)
		NCT02569398 (II/III)			
Dose	12, 40 or 60 mg	10, 25 or 50 mg	20 or 50 mg	25, 50, 100 and 200 mg	1, 10, 25 and 75 mg
Trial duration	78, 104 and 260 weeks	26, 52 or 96 weeks or 54 months	97 or 104 weeks	18 months	13 weeks and 5 years
1^st^ Outcomes	Baseline change in ADAS-Cog and ADCS-ADL scores	Safety measure by AEs or SAEs up to 10 months; Baseline change in ADCS-PACC Score (54 weeks),	Baseline change in CDR-SB Score	Baseline change in ADCOMS, Safety measure by AEs or SAEs	Safety measure by AEs or SAEs
2^nd^ Outcomes	Baseline change in CDR-SB score; CSF tau, brain amyloid load, NPI score, hippocampal volume, MMSE, score, etc.	Baseline change in CSF and plasma Aβ37, Aβ38, Aβ40, Aβ42, sAPPα, sAPPβ, Tau; CFI; CDR-SB, Neurodegeneration by Assessing Changes in Imaging Biomarkers, etc.	Baseline change in ADAS-Cog-13 score; CSF Aβ40, Aβ42, Tau; brain amyloid load by PET imaging, while brain volume, NPI score; ADCS-ADL score, etc.	volumetric Magnetic Resonance Imaging; Baseline change in CSF and plasma Aβ1-x, etc.	Baseline change in CDR-SB score; CSF Aβ40, Aβ42, total tau and phosphorylated tau, volumetric MRI; Everyday Cognition scale, etc.

More detailed measures of outcomes are listed under each trail protocol in the relevant website

Abbreviations used in the table: *ADAS-Cog-13 Score* Alzheimer’s disease assessment scale- cognitive subscale score, *ADCOMS* Alzheimer’s disease composite score, *ADCS-ADL* Alzheimer’s disease cooperative study activities of daily living inventory instrumental items score, *ADCS-PACC Score* Alzheimer’s disease cooperative study preclinical alzheimer cognitive composite score, *CDR Score* change in clinical Dementia rating global score, *CDR-SB Score* the clinical Dementia rating - sum of boxes score, *CFI* cognitive function index, *FAQ Score* functional activities questionnaire score, *MMSE Score* mini-mental state examination score, *NPI Score* neuropsychiatric inventory score, Safety and tolerability by assessing adverse events (AEs) and serious adverse events (SAEs)

### AZD-3839 and AZD-3293

**Fig. 2:**
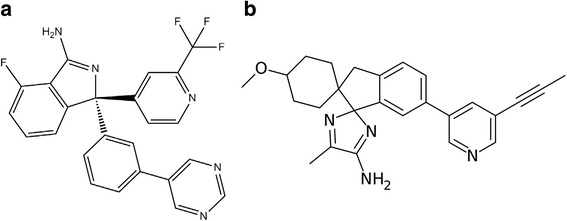
Chemical structures of compound AZD-3293. **a** AZD-3289 is a potent BACE1 inhibitor with a chemical formula of C_26_H_28_N_4_O and a structure name of (3S)-3-[2-(difluoromethyl)pyridin-4-yl]-7-fluoro-3-(3-pyrimidin-5-ylphenyl)-1,2-dihydroisoindol-1-amine. It’s molecular weight is 449. **b** AZD-3293 is developed at AstraZeneca and Astex with a chemical formula of C_26_H_28_N_4_O and structural name of 4-Methoxy-5′′-methyl-6′-[5-(prop-1-yn-1-yl)pyridin-3-yl]-3′H-dispiro[cyclohexane-1,2′-indene-1′,2′′-imidazole]-4′′-amine. It’s molecular weight is 412. 54

AstraZeneca is another leading pharmaceutical company engaged in the identification of 6-substituted isocytosine as a novel compound against BACE1 through fragment-based 1D NMR screen together with characterization by BIAcore and co-crystalization [21]. This initial fragment hit with an isocytosine core has mM potency. By NMR affinity screening and structure-guided evolution, together with X-ray crystallography and potency determination using surface plasmon resonance, a series of compounds with the dihydroisocytosine scaffold were found to be more potent through functional enzyme inhibition assays [14]. The most potent compound in this series has an IC_50_ of 80 nM for BACE1 inhibition. Using a scaffold hopping protocol from dihydroisocytosine to aminohydantoin, the potent compound AZD-3839 (Fig. 2a), with an IC_50_ of 4.8 nM for Aβ40 reduction and an IC_50_ of 16.7 nM for sAPPβ reduction, was finally developed [78]. AZD-3839 has 14 and >1000-fold greater selectivity over BACE2 and cathepsin D, respectively, and orally administrated AZD-3839 shows dose- and time-dependent lowering of plasma, brain, and CSF Aβ40, Aβ42, and sAPPβ levels in mouse (C57BL/6 WT mice), guinea pig, and non-human primate [42]. Interestingly, treatment with this compound showed a biphasic effect, which causes an initial small increase in Aβ levels followed by a subsequent significant reduction in Aβ, similar to our previous reports for γ-secretase inhibition [7]. It has also been reported that the IC_50_ values to reduce Aβ40 in plasma were 64- and 48-fold lower than those in brains for mouse and guinea pig, respectively [42]. Based on the overall pharmacological profile and its drug-like properties shown in animal studies, AZD-3839 was advanced to Phase 1 clinical trials in May 2011 in the UK, but these were terminated in December, 2012. It is not known whether this was related to the high affinity of AZD-3839 for the human ether-a-go-go related gene (hERG) ion channel as previously reported [78].

Despite this setback, AstraZeneca launched multiple phase I trials on another compound AZD-3293 (see structure in Fig. 2b) in December, 2012. AZD-3293 was licensed from Astex Pharmaceuticals as a potent BACE1 inhibitor with a [K_i_] of 0.4 nM by an enzymatic TR-FRET assay [15]. In this report, it showed inhibition of Aβ40 and sAPPβ in the pM range in SH-SY5Y cells over-expressing AβPP (IC_50_  =  610, 310, and 80 pM, respectively), and showed nearly equal potency against BACE2 but a >25,000- and >41,000-fold selectivity against cathepsin D and γ-secretase cleavage of Notch IC_50_ values, respectively. The IC_50_ for brain Aβ_40_ reduction was estimated to be 600 pM in mice, 900 pmol/L (CV 4 %) in guinea pigs, and 800 pmol/L (CV 9 %) in dog. Based on free AZD-3293 concentration in the CSF, the IC_50_ for Aβ_40_ reduction is 3.8 nmol/L (CV 31 %). The affinity of AZD-3293 on hERG was also investigated by the whole-cell patch clamp technique using hERG-expressing CHO cells and the IC_50_ for the inhibition of hERG is over 33 μM.

The encouraging results in animals are apparently correlated with human studies. In the 2014 13th International Geneva/Springfield Symposium on Advances in Alzheimer Therapy, AstraZeneca reported successful phase I trial results from 72 healthy volunteers, which showed a reduction of CSF Aβ as much as 75 % that requires 2 to 3 weeks after treatment to return to baseline levels (http://www.alzforum.org/news/research-news/bace-inhibitor-heads-phase-23-trials).

In September 2014, AstraZeneca and Eli Lilly announced joint development of AZD-3293 and the implementation of a pivotal Phase II/III trial enrolling 2202 patients selected by multiple NIA-AA criteria for MCI due to AD or mild AD and confirmed by either an amyloid PET scan or a lumbar puncture (http://www.alzforum.org/therapeutics/azd3293). This 5-year trial will compare two doses given once daily as a tablet to placebo and will measure success by changes from baseline on the clinical dementia rating sum of boxes (CDR-SOB) (Table 1). The ADAS-cog and ADCS-ADL are secondary outcome measures, along with other clinical markers as well as change in CSF markers, functional and amyloid PET, and MRI.

To test potential interactions of AZD-3293 with commonly prescribed drugs such as the blood thinners warfarin and dabigatran, the sedative midalozam, a lipid-lowering medication simvastatin (Zocor) as well as inhibitor of cholinesterases donepezil (Aricept), AstraZeneca added three additional phase I trials in a total of 157 healthy volunteers in 2015. No results have been report thus far.

### JNJ-54861911

In March 2013, Janssen began Phase I trials of JNJ-54861911, which was initially developed by and licensed from Shionogi & Co. in Japan and developed through the collaboration between two companies. JNJ-54861911 was optimized through multiple steps by the Shionogi group from hits with amino-dihydrothiazine or modified versions of the cyclic isothiourea warhead [63]. By the cyclopropane-based conformational restriction approach, replacing an amidine group with chiral cyclopropane rings, or adding an amide linker between the two aryl rings as exemplified, multiple compound series were explored [97–99]. These efforts eventually yielded more potent and bioavailable JNJ-54861911 (structures have not been revealed yet). Phase I trial participants were given various doses (1, 3, 9, 27, 81, and 160 mg in healthy volunteers or 5, 10, 25, and 90 mg in elderly volunteers) of JNJ-54861911 or placebo once daily for 14 days to 4 weeks. In an AD/PD conference held in Nice in 2015, Dr. Johannes Streffer from Janssen reported that those taking the drug showed safe profiles in 94 people. The compound crossed the blood-brain barrier and displayed dose-dependent reduction of all four forms of Aβ peptides (Aβ37, Aβ38, Aβ40, and Aβ42). A dose of 25 mg showed reduction of CSF Aβ concentrations by 80 %, and up to 95 % Aβ reduction was achieved with once-daily oral dosing of 90 mg. Reduction of the BACE cleavage product sAPPβ correlated with reduction of Aβ, whereas levels of sAPPα rose by 2- to 2.5-fold, a method first reported for assessing BACE1 activity [92].

In October 2015, a phase IIb/III randomized, double-blind, Placebo-Controlled trial were launched in Europe, Australia, and Mexico. The study is expected to enroll 1650 individuals who are asymptomatic but at risk for developing Alzheimer’s dementia (Table 1). The trial will investigate the efficacy and safety of JNJ-54861911. A dose of either 10 or 25 mg of drug will be given for 54 months for end-point evaluation. In January, 2016, Janssen added a phase I study in 32 healthy adults in Germany to evaluate drug interactions between JNJ-54861911 and the anti-diabetic metformin or rosuvastatin (also called Crestor for cholesterol reduction).

### E2609

Although not explicitly described, Eisai may have also identified its potent BACE1 inhibitor E2609 through FDBB screening. In his review, Oehlrich implied that Eisai developed its BACE1 inhibitor through several series of bicyclic aminodihydrothiazines fused with unsaturated five- and six-membered rings, eventually culminating in a structure similar to a Merck BACE1 inhibitor, with a Ki of 27 nM [63]. Significant reductions in Aβ levels in the CSF and plasma in nonhuman primates were demonstrated in preclinical animal testing (poster presentation by Fukushima T, Lucas F at the 2012 Alzheimer’s Association International Conference). In two standard phase I trials, 73 healthy adult individuals received single doses of E2609 between 5 and 800 mg (divided into nine cohorts), while another 50 individuals received E2609 doses between 25 and 400 mg daily for 14 days. The drug E2609 showed tolerability at all doses tested for single use, with the most common adverse events included headache and dizziness. Individuals receiving repeated doses up to 200 mg had no clinically significant safety concerns. In both cases, plasma Aβ [Aβ(1-x)] levels were measured prior to patients receiving E2609. The maximum dose-dependent reduction of plasma Aβ(1-X) relative to baseline was 52 % at 5 mg and 92 % at 800 mg. The percentage decrease in CSF Aβ(1-x) after 14 days dosing compared to baseline was statistically significant, showing a 46.2, 61.9, 73.8, and 79.9 % reduction compared to placebo in the 25, 50, 100, and 200 mg cohorts, respectively. In November, 2014, these encouraging results led Eisai to announce a large phase II dose-finding study by collaborating with Biogen. It will enroll 700 people with MCI due to AD or prodromal AD and being amyloid PET-scan positive.

### CNP520

In late 2015, Novartis announced a global collaboration with Amgen to test a BACE inhibitor, CNP520, in human trials and to develop further compounds from the pre-clinical BACE inhibitor programs of both companies. Novartis’ CNP520 is formulated to be taken as capsules for oral administration and has gone through preclinical studies in the company, although little information on the potency and structure of this drug has been disclosed. It is expected to have efficacy high enough for moving the compound into multi-site phase I/IIa trials in the Netherlands, Belgium, Germany, the UK, and the USA (http://www.alzforum.org/therapeutics/cnp520). Doses of CNP520 ranging from 1, 10, 25, and 75 mg will be given to 125 healthy individuals between 60 and 80 years of age once daily for 13 weeks and Aβ38, 40, and 42 will be measured at the end of trials for comparison to baseline levels.

The unique part of Norvatis’ Phase II/III trial, announced in November, 2015, is to test two investigational drugs, CNP520 and CAD106. CAD106 was developed at Novartis for active vaccination using multiple copies of Aβ1-6 peptide derived from the N-terminal B cell epitope of Aβ (http://www.alzforum.org/therapeutics/cad106). Five phase II trials involving a total of 274 people were concluded in 2014, and no major safety concerns in individuals administrated CAD106 for 55 to 66 weeks were reported, while induction of Aβ-specific antibody was evident [17]. In combining with this Aβ-clearance strategy, administration of CNP520, administered separately, is expected to stop generation of Aβ and to remove existing Aβ plaques. This combination of trials will enroll 1340 homozygous ApoE4 carriers between the ages of 60 and 75. In this 5-year trial, about half of the participants will be injected with CAD106 intramuscularly at weeks 1, 7, 13, 24 and then quarterly randomized to compare to matching placebos. The other half will be randomized to compare once-daily CNP520 to matching placebos. The trial results will not likely be released until 2023.

Norvatis also developed another compound named NB-360, which shows potent inhibition of BACE1 in APP transgenic mice, rat and pigs [61], but this compound has not yet been developed for clinical trial.

## Conclusions

Over the past decade, substantial research efforts have been directed toward understanding BACE1 as a critical target for AD therapy. Although FBDD-based hits have been successful in advancing BACE1 inhibitors to clinical trials, discovery of BACE1 inhibitors by HTS or *in silico* designs should not be discounted, as they also yielded hits that were further optimized to increase potency at Wyeth and Roche. For example, HTS identified amidine-based pharmacophore, which was independently discovered by FBDD, showing an important convergence in drug discovery.

While there is clear evidence that brain-penetrable BACE1 inhibitors reduce Aβ generation and likely amyloid deposition, the safety profiles for long-term use of these inhibitors should be closely monitored. Studies based on the characterizations of BACE1-null mice have revealed roles of BACE1 in myelination, ion channel activities, neuronal migration and excitation, and astrogenesis [see recent reviews by [4, 35, 43, 89, 95]]. In addition to potential on-site toxicity, many promising drugs are doomed by having certain off-site toxicity, and any intolerable toxicity will prevent the further application of some drugs in humans. Safety fears have prompted termination of several BACE1 inhibitors in trials. LY-2811376 and LY-2886721 from Eli Lilly, AZD-3839 from AstraZeneca, and RG-7129 from Roche were all terminated during clinical trials. While both compounds from Eli Lilly were reported to cause unacceptable side effects (due to evidence of liver toxicity in patients), the reasons for the termination of the trials involving the latter two drugs were not disclosed. Another orally active BACE1 inhibitor, BI1181181/VTP-37948, discovered by Vitae Pharmaceutics and developed by Boehringer Ingelheim, finished two Phase 1 clinical trials and demonstrated strong potency in reducing brain Aβ in volunteers in October of 2014. Surprisingly, this compound was not advanced to further clinical trials by these two ventured companies. In fact, Boehringer has terminated development of BI1181181 in July 2015, likely related to concerns regarding side effects of this experimental BACE1 inhibitor drug. Hence, placing safe BACE1 inhibitory drugs on the market remains challenging, despite the great promise shown in several trials.

Despite the above concerns and challenges, it should be recognized that BACE1 is perhaps the best target for reducing Aβ generation, considering the relatively mild phenotypes exhibited by BACE1-null mice. This may be related to the fact that most BACE1 substrates can also be complementarily processed by α-secretase for ectodomain shedding. Indeed, after the initial reports of the BACE1 sequence in late 1999, drug discovery efforts in blocking BACE1 activity in humans have never been abandoned, no matter how many challenges were presented over the past 17 years. There is great anticipation that optimal inhibition of BACE1 in humans will eventually answer whether a so-called “amyloid hypothesis”-based strategy is the best way to improve cognitive function in AD patients.

## References

[1] Albert JS (2009). Progress in the development of beta-secretase inhibitors for Alzheimer’s disease. Prog Med Chem.

[2] Alcarraz-Vizan G, Casini P, Cadavez L, Visa M, Montane J, Servitja JM, Novials A (2015). Inhibition of BACE2 counteracts hIAPP-induced insulin secretory defects in pancreatic beta-cells. FASEB J.

[3] Barao S, Gartner A, Leyva-Diaz E, Demyanenko G, Munck S, Vanhoutvin T, Zhou L, Schachner M, Lopez-Bendito G, Maness PF, De SB (2015). Antagonistic Effects of BACE1 and APH1B-gamma-Secretase Control Axonal Guidance by Regulating Growth Cone Collapse. Cell Rep.

[4] Barao S, Moechars D, Lichtenthaler SF, De SB (2016). BACE1 Physiological Functions May Limit Its Use as Therapeutic Target for Alzheimer’s Disease. Trends Neurosci.

[5] Braak H, Braak E (1997). Diagnostic criteria for neuropathologic assessment of Alzheimer’s disease. Neurobiol Aging.

[6] Cai H, Wang Y, McCarthy D, Wen H, Borchelt DR, Price DL, Wong PC (2001). BACE1 is the major beta-secretase for generation of Abeta peptides by neurons. Nat Neurosci.

[7] Carter DB, Dunn E, Pauley AM, McKinley DD, Fleck TJ, Ellerbrook BR, Stratman NC, Zhou X, Himes CS, Nye JS, Tomasselli A, Yan R (2008). Changes in gamma-secretase activity and specificity caused by the introduction of consensus aspartyl protease active motif in Presenilin 1. Mol Neurodegener.

[8] Casas S, Casini P, Piquer S, Altirriba J, Soty M, Cadavez L, Gomis R, Novials A (2010). BACE2 plays a role in the insulin receptor trafficking in pancreatic beta-cells. Am J Physiol Endocrinol Metab.

[9] Cheret C, Willem M, Fricker FR, Wende H, Wulf-Goldenberg A, Tahirovic S, Nave KA, Saftig P, Haass C, Garratt AN, Bennett DL, Birchmeier C (2013). Bace1 and Neuregulin-1 cooperate to control formation and maintenance of muscle spindles. EMBO J.

[10] Das S, Chakraborty S, Basu S (2015). Fragment-based designing for the generation of novel leads against BACE1. Cent Nerv Syst Agents Med Chem.

[11] De Strooper B, Saftig P, Craessaerts K, Vanderstichele H, Guhde G, Annaert W, Von Figura K, Van Leuven F (1998). Deficiency of presenilin-1 inhibits the normal cleavage of amyloid precursor protein. Nature.

[12] De SB, Vassar R, Golde T (2010). The secretases: enzymes with therapeutic potential in Alzheimer disease. Nat Rev Neurol.

[13] Dominguez D, Tournoy J, Hartmann D, Huth T, Cryns K, Deforce S, Serneels L, Camacho IE, Marjaux E, Craessaerts K, Roebroek AJ, Schwake M, D’Hooge R, Bach P, Kalinke U, Moechars D, Alzheimer C, Reiss K, Saftig P, De Strooper B (2005). Phenotypical and biochemical analysis of BACE1 and BACE2 deficient mice. J Biol Chem.

[14] Edwards PD (2007). Application of fragment-based lead generation to the discovery of novel, cyclic amidine beta-secretase inhibitors with nanomolar potency, cellular activity, and high ligand efficiency. J Med Chem.

[15] Eketjall S, Janson J, Kaspersson K, Bogstedt A, Jeppsson F, Falting J, Haeberlein SB, Kugler AR, Alexander RC, Cebers G (2016). AZD3293: A Novel, Orally Active BACE1 Inhibitor with High Potency and Permeability and Markedly Slow Off-Rate Kinetics. J Alzheimers Dis.

[16] Esterhazy D, Stutzer I, Wang H, Rechsteiner MP, Beauchamp J, Dobeli H, Hilpert H, Matile H, Prummer M, Schmidt A, Lieske N, Boehm B, Marselli L, Bosco D, Kerr-Conte J, Aebersold R, Spinas GA, Moch H, Migliorini C, Stoffel M (2011). Bace2 is a beta cell-enriched protease that regulates pancreatic beta cell function and mass. Cell Metab.

[17] Farlow MR, Andreasen N, Riviere ME, Vostiar I, Vitaliti A, Sovago J, Caputo A, Winblad B, Graf A (2015). Long-term treatment with active Abeta immunotherapy with CAD106 in mild Alzheimer’s disease. Alzheimers Res Ther.

[18] Farzan M, Schnitzler CE, Vasilieva N, Leung D, Choe H (2000). BACE2, a beta -secretase homolog, cleaves at the beta site and within the amyloid-beta region of the amyloid-beta precursor protein. Proc Natl Acad Sci U S A.

[19] Forman, M., H. Kleijn, M. Dockendorf, J. Palcza, J. Tseng, C. Canales, M. Egan, M. Kennedy, O. Laterza, L. Ma, J. Scott, M. Tanen, J. Apter, M. Backonja, L. Ereshefsky, H. Gevorkyan, S. Jhee, R. Rynders, A. Zari, E. Bryan, J. Wagner, M. Troyer, and J. Stone. 2013. The novel BACE inhibitor MK-8931 dramatically lowers CSF beta-amyloid in patients with mild-to-moderate Alzheimer’s disease. Alzheimer's & Dementia: The Journal of the Alzheimer's Association 9(Supplement):P139.

[20] Francis R, McGrath G, Zhang J, Ruddy DA, Sym M, Apfeld J, Nicoll M, Maxwell M, Hai B, Ellis MC, Parks AL, Xu W, Li J, Gurney M, Myers RL, Himes CS, Hiebsch R, Ruble C, Nye JS, Curtis D (2002). aph-1 and pen-2 are required for Notch pathway signaling, gamma-secretase cleavage of betaAPP, and presenilin protein accumulation. Dev Cell.

[21] Geschwindner S, Olsson LL, Albert JS, Deinum J, Edwards PD, de Beer T, Folmer RH (2007). Discovery of a novel warhead against beta-secretase through fragment-based lead generation. J Med Chem.

[22] Ghosh AK, Brindisi M, Tang J (2012). Developing beta-secretase inhibitors for treatment of Alzheimer’s disease. J Neurochem.

[23] Ghosh AK, Osswald HL (2014). BACE1 (beta-secretase) inhibitors for the treatment of Alzheimer’s disease. Chem Soc Rev.

[24] Golde TE, Dickson D, Hutton M (2006). Filling the gaps in the abeta cascade hypothesis of Alzheimer’s disease. Curr Alzheimer Res.

[25] Gotz J, Chen F, van Dorpe J, Nitsch RM (2001). Formation of neurofibrillary tangles in P301l tau transgenic mice induced by Abeta 42 fibrils. Science.

[26] Grundke-Iqbal I, Iqbal K, Quinlan M, Tung YC, Zaidi MS, Wisniewski HM (1986). Microtubule-associated protein tau. A component of Alzheimer paired helical filaments. J Biol Chem.

[27] Haas C (2012). Strategies, development, and pitfalls of therapeutic options for Alzheimer’s disease. J Alzheimers Dis.

[28] Haass C (2004). Take five-BACE and the gamma-secretase quartet conduct Alzheimer’s amyloid beta-peptide generation. EMBO J.

[29] Hardy J, Selkoe DJ (2002). The amyloid hypothesis of Alzheimer’s disease: progress and problems on the road to therapeutics. Science.

[30] Hilpert H (2013). Beta-Secretase (BACE1) inhibitors with high in vivo efficacy suitable for clinical evaluation in Alzheimer’s disease. J Med Chem.

[31] Hitt B, Riordan SM, Kukreja L, Eimer WA, Rajapaksha TW, Vassar R (2012). Beta-Site amyloid precursor protein (APP)-cleaving enzyme 1 (BACE1)-deficient mice exhibit a close homolog of L1 (CHL1) loss-of-function phenotype involving axon guidance defects. J Biol Chem.

[32] Hitt BD, Jaramillo TC, Chetkovich DM, Vassar R (2010). BACE1−/− mice exhibit seizure activity that does not correlate with sodium channel level or axonal localization. Mol Neurodegener.

[33] Hong L, Koelsch G, Lin X, Wu S, Terzyan S, Ghosh AK, Zhang XC, Tang J (2000). Structure of the protease domain of memapsin 2 (beta-secretase) complexed with inhibitor. Science.

[34] Hong L, Turner RT, Koelsch G, Shin D, Ghosh AK, Tang J (2002). Crystal structure of memapsin 2 (beta-secretase) in complex with an inhibitor OM00-3. Biochemistry.

[35] Hu X, Fan Q, Hou H, Yan R. Neurological dysfunctions associated with altered BACE1-dependent Neuregulin-1 signaling. J Neurochem. 2015. [Epub ahead of print] 10.1111/jnc.13395 PMC4833723 26465092

[36] Hu X, He W, Diaconu C, Tang X, Kidd GJ, Macklin WB, Trapp BD, Yan R (2008). Genetic deletion of BACE1 in mice affects remyelination of sciatic nerves. FASEB J.

[37] Hu X, He W, Luo X, Tsubota KE, Yan R (2013). BACE1 regulates hippocampal astrogenesis via the Jagged1-Notch pathway. Cell Rep.

[38] Hu X, Hicks CW, He W, Wong P, Macklin WB, Trapp BD, Yan R (2006). Bace1 modulates myelination in the central and peripheral nervous system. Nat Neurosci.

[39] Hu X, Li X, Zhao M, Gottesdiener A, Luo W, Paul S (2014). Tau pathogenesis is promoted by Abeta1-42 but not Abeta1-40. Mol Neurodegener.

[40] Hu X, Zhou X, He W, Yang J, Xiong W, Wong P, Wilson CG, Yan R (2010). BACE1 deficiency causes altered neuronal activity and neurodegeneration. J Neurosci.

[41] Hussain I, Powell D, Howlett DR, Tew DG, Meek TD, Chapman C, Gloger IS, Murphy KE, Southan CD, Ryan DM, Smith TS, Simmons DL, Walsh FS, Dingwall C, Christie G (1999). Identification of a novel aspartic protease (Asp 2) as beta-secretase. Mol Cell Neurosci.

[42] Jeppsson F, Eketjall S, Janson J, Karlstrom S, Gustavsson S, Olsson LL, Radesater AC, Ploeger B, Cebers G, Kolmodin K, Swahn BM, von Berg S, Bueters T, Falting J (2012). Discovery of AZD3839, a potent and selective BACE1 inhibitor clinical candidate for the treatment of Alzheimer disease. J Biol Chem.

[43] Kandalepas PC, Vassar R (2014). The normal and pathologic roles of the Alzheimer’s beta-secretase, BACE1. Curr Alzheimer Res.

[44] Kimura T, Shuto D, Hamada Y, Igawa N, Kasai S, Liu P, Hidaka K, Hamada T, Hayashi Y, Kiso Y (2005). Design and synthesis of highly active Alzheimer’s beta-secretase (BACE1) inhibitors, KMI-420 and KMI-429, with enhanced chemical stability. Bioorg Med Chem Lett.

[45] Kimura T, Shuto D, Kasai S, Liu P, Hidaka K, Hamada T, Hayashi Y, Hattori C, Asai M, Kitazume S, Saido TC, Ishiura S, Kiso Y (2004). KMI-358 and KMI-370, highly potent and small-sized BACE1 inhibitors containing phenylnorstatine. Bioorg Med Chem Lett.

[46] Koike M, Nakanishi H, Saftig P, Ezaki J, Isahara K, Ohsawa Y, Schulz-Schaeffer W, Watanabe T, Waguri S, Kametaka S, Shibata M, Yamamoto K, Kominami E, Peters C, von Figura K, Uchiyama Y (2000). Cathepsin D deficiency induces lysosomal storage with ceroid lipofuscin in mouse CNS neurons. J Neurosci.

[47] Kosik KS, Joachim CL, Selkoe DJ (1986). Microtubule-associated protein tau (tau) is a major antigenic component of paired helical filaments in Alzheimer disease. Proc Natl Acad Sci U S A.

[48] Kowall NW, Kosik KS (1987). Axonal disruption and aberrant localization of tau protein characterize the neuropil pathology of Alzheimer’s disease. Ann Neurol.

[49] Kuhn PH, Koroniak K, Hogl S, Colombo A, Zeitschel U, Willem M, Volbracht C, Schepers U, Imhof A, Hoffmeister A, Haass C, Rossner S, Brase S, Lichtenthaler SF (2012). Secretome protein enrichment identifies physiological BACE1 protease substrates in neurons. EMBO J.

[50] LaFerla FM, Oddo S (2005). Alzheimer’s disease: Abeta, tau and synaptic dysfunction. Trends Mol Med.

[51] Lewis J, Dickson DW, Lin WL, Chisholm L, Corral A, Jones G, Yen SH, Sahara N, Skipper L, Yager D, Eckman C, Hardy J, Hutton M, McGowan E (2001). Enhanced neurofibrillary degeneration in transgenic mice expressing mutant tau and APP. Science.

[52] Li Y, Bohm C, Dodd R, Chen F, Qamar S, Schmitt-Ulms G, Fraser PE, St George-Hyslop PH (2014). Structural biology of presenilin 1 complexes. Mol Neurodegener.

[53] Li YM, Lai MT, Xu M, Huang Q, DiMuzio-Mower J, Sardana MK, Shi XP, Yin KC, Shafer JA, Gardell SJ (2000). Presenilin 1 is linked with gamma-secretase activity in the detergent solubilized state. Proc Natl Acad Sci U S A.

[54] Lin X, Koelsch G, Wu S, Downs D, Dashti A, Tang J (2000). Human aspartic protease memapsin 2 cleaves the beta-secretase site of beta-amyloid precursor protein. Proc Natl Acad Sci U S A.

[55] Luo X, Yan R (2010). Inhibition of BACE1 for therapeutic usein Alzheimer’s disease. Int J Clin Exp Pathol.

[56] Luo Y, Bolon B, Kahn S, Bennett BD, Babu-Khan S, Denis P, Fan W, Kha H, Zhang J, Gong Y, Martin L, Louis JC, Yan Q, Richards WG, Citron M, Vassar R (2001). Mice deficient in BACE1, the Alzheimer’s beta-secretase, have normal phenotype and abolished beta-amyloid generation. Nat Neurosci.

[57] Malamas MS, Erdei J, Gunawan I, Barnes K, Johnson M, Hui Y, Turner J, Hu Y, Wagner E, Fan K, Olland A, Bard J, Robichaud AJ (2009). Aminoimidazoles as potent and selective human beta-secretase (BACE1) inhibitors. J Med Chem.

[58] Mandal M (2016). Structure-Based Design of an Iminoheterocyclic beta-Site Amyloid Precursor Protein Cleaving Enzyme (BACE) Inhibitor that Lowers Central Abeta in Nonhuman Primates. J Med Chem.

[59] Masters CL, Beyreuther K (2006). Alzheimer’s centennial legacy: prospects for rational therapeutic intervention targeting the Abeta amyloid pathway. Brain.

[60] Musiek ES, Holtzman DM (2015). Three dimensions of the amyloid hypothesis: time, space and ‘wingmen’. Nat Neurosci.

[61] Neumann U, Rueeger H, Machauer R, Veenstra SJ, Lueoend RM, Tintelnot-Blomley M, Laue G, Beltz K, Vogg B, Schmid P, Frieauff W, Shimshek DR, Staufenbiel M, Jacobson LH (2015). A novel BACE inhibitor NB-360 shows a superior pharmacological profile and robust reduction of amyloid-beta and neuroinflammation in APP transgenic mice. Mol Neurodegener.

[62] Oddo S, Caccamo A, Shepherd JD, Murphy MP, Golde TE, Kayed R, Metherate R, Mattson MP, Akbari Y, LaFerla FM (2003). Triple-transgenic model of Alzheimer’s disease with plaques and tangles: intracellular Abeta and synaptic dysfunction. Neuron.

[63] Oehlrich D, Prokopcova H, Gijsen HJ (2014). The evolution of amidine-based brain penetrant BACE1 inhibitors. Bioorg Med Chem Lett.

[64] Price JL, Morris JC (1999). Tangles and plaques in nondemented aging and “preclinical” Alzheimer’s disease. Ann Neurol.

[65] Rajapakse HA (2006). Discovery of oxadiazoyl tertiary carbinamine inhibitors of beta-secretase (BACE-1). J Med Chem.

[66] Roberds SL (2001). BACE knockout mice are healthy despite lacking the primary beta-secretase activity in brain: implications for Alzheimer’s disease therapeutics. Hum Mol Genet.

[67] Rochin L, Hurbain I, Serneels L, Fort C, Watt B, Leblanc P, Marks MS, De SB, Raposo G, van Neil G (2013). BACE2 processes PMEL to form the melanosome amyloid matrix in pigment cells. Proc Natl Acad Sci U S A.

[68] Selkoe DJ (2002). Alzheimer’s disease is a synaptic failure. Science.

[69] Selkoe DJ (2004). Alzheimer disease: mechanistic understanding predicts novel therapies. Ann Intern Med.

[70] Shacka JJ, Klocke BJ, Young C, Shibata M, Olney JW, Uchiyama Y, Saftig P, Roth KA (2007). Cathepsin D deficiency induces persistent neurodegeneration in the absence of Bax-dependent apoptosis. J Neurosci.

[71] Sinha S (1999). Purification and cloning of amyloid precursor protein beta-secretase from human brain. Nature.

[72] Sisodia SS, George-Hyslop PH (2002). gamma-Secretase, Notch, Abeta and Alzheimer’s disease: where do the presenilins fit in?. Nat Rev Neurosci.

[73] Southan C, Hancock JM (2013). A tale of two drug targets: the evolutionary history of BACE1 and BACE2. Front Genet.

[74] Stachel SJ (2004). Structure-based design of potent and selective cell-permeable inhibitors of human beta-secretase (BACE-1). J Med Chem.

[75] Stamford A, Strickland C (2013). Inhibitors of BACE for treating Alzheimer’s disease: a fragment-based drug discovery story. Curr Opin Chem Biol.

[76] Stamford AW (2012). Discovery of an Orally Available, Brain Penetrant BACE1 Inhibitor that Affords Robust CNS Abeta Reduction. ACS Med Chem Lett.

[77] Sun X, He G, Song W (2006). BACE2, as a novel APP theta-secretase, is not responsible for the pathogenesis of Alzheimer’s disease in Down syndrome. FASEB J.

[78] Swahn BM (2012). Design and synthesis of beta-site amyloid precursor protein cleaving enzyme (BACE1) inhibitors with in vivo brain reduction of beta-amyloid peptides. J Med Chem.

[79] Szecsi PB (1992). The aspartic proteases. Scand J Clin Lab Invest Suppl.

[80] Tamamura H, Kato T, Otaka A, Fujii N (2003). Synthesis of potent beta-secretase inhibitors containing a hydroxyethylamine dipeptide isostere and their structure-activity relationship studies. Org Biomol Chem.

[81] Tang J, Ghosh AK, Hong L, Koelsch G, Turner RT, Chang W (2003). Study of memapsin 2 (beta-secretase) and strategy of inhibitor design. J Mol Neurosci.

[82] Tang J, Lin X (1994). Engineering aspartic proteases to probe structure and function relationships. Curr Opin Biotechnol.

[83] Trojanowski JQ, Lee VM (2001). Brain degeneration linked to “fatal attractions” of proteins in Alzheimer’s disease and related disorders. J Alzheimers Dis.

[84] Tsukuba T, Okamoto K, Okamoto Y, Yanagawa M, Kohmura K, Yasuda Y, Uchi H, Nakahara T, Furue M, Nakayama K, Kadowaki T, Yamamoto K, Nakayama KI (2003). Association of cathepsin E deficiency with development of atopic dermatitis. J Biochem.

[85] Tu S, Okamoto S, Lipton SA, Xu H (2014). Oligomeric Abeta-induced synaptic dysfunction in Alzheimer’s disease. Mol Neurodegener.

[86] van Bebber F, Hruscha A, Willem M, Schmid B, Haass C (2013). Loss of Bace2 in zebrafish affects melanocyte migration and is distinct from Bace1 knock out phenotypes. J Neurochem.

[87] Vassar R (2014). BACE1 inhibitor drugs in clinical trials for Alzheimer’s disease. Alzheimers Res Ther.

[88] Vassar R (1999). Beta-secretase cleavage of Alzheimer’s amyloid precursor protein by the transmembrane aspartic protease BACE. Science.

[89] Vassar R, Kuhn PH, Haass C, Kennedy ME, Rajendran L, Wong PC, Lichtenthaler SF (2014). Function, therapeutic potential and cell biology of BACE proteases: current status and future prospects. J Neurochem.

[90] Wang YS, Strickland C, Voigt JH, Kennedy ME, Beyer BM, Senior MM, Smith EM, Nechuta TL, Madison VS, Czarniecki M, McKittrick BA, Stamford AW, Parker EM, Hunter JC, Greenlee WJ, Wyss DF (2010). Application of fragment-based NMR screening, X-ray crystallography, structure-based design, and focused chemical library design to identify novel microM leads for the development of nM BACE-1 (beta-site APP cleaving enzyme 1) inhibitors. J Med Chem.

[91] Willem M, Garratt AN, Novak B, Citron M, Kaufmann S, Rittger A, DeStrooper B, Saftig P, Birchmeier C, Haass C (2006). Control of peripheral nerve myelination by the beta-secretase BACE1. Science.

[92] Yan R, Bienkowski MJ, Shuck ME, Miao H, Tory MC, Pauley AM, Brashier JR, Stratman NC, Mathews WR, Buhl AE, Carter DB, Tomasselli AG, Parodi LA, Heinrikson RL, Gurney ME (1999). Membrane-anchored aspartyl protease with Alzheimer’s disease beta-secretase activity. Nature.

[93] Yan R, Fan Q, Zhou J, Vassar R (2016). Inhibiting BACE1 to reverse synaptic dysfunctions in Alzheimer’s disease. Neurosci Biobehav Rev.

[94] Yan R, Munzner JB, Shuck ME, Bienkowski MJ (2001). BACE2 functions as an alternative alpha-secretase in cells. J Biol Chem.

[95] Yan R, Vassar R (2014). Targeting the beta secretase BACE1 for Alzheimer’s disease therapy. Lancet Neurol.

[96] Yanagawa M, Tsukuba T, Nishioku T, Okamoto Y, Okamoto K, Takii R, Terada Y, Nakayama KI, Kadowaki T, Yamamoto K (2007). Cathepsin E deficiency induces a novel form of lysosomal storage disorder showing the accumulation of lysosomal membrane sialoglycoproteins and the elevation of lysosomal pH in macrophages. J Biol Chem.

[97] Yonezawa S, Fujiwara K, Yamamoto T, Hattori K, Yamakawa H, Muto C, Hosono M, Tanaka Y, Nakano T, Takemoto H, Arisawa M, Shuto S (2013). Conformational restriction approach to beta-secretase (BACE1) inhibitors III: effective investigation of the binding mode by combinational use of X-ray analysis, isothermal titration calorimetry and theoretical calculations. Bioorg Med Chem.

[98] Yonezawa S, Yamakawa H, Muto C, Hosono M, Yamamoto T, Hattori K, Sakagami M, Togame H, Tanaka Y, Nakano T, Takemoto H, Arisawa M, Shuto S (2013). Conformational restriction approach to BACE1 inhibitors II: SAR study of the isocytosine derivatives fixed with a cis-cyclopropane ring. Bioorg Med Chem Lett.

[99] Yonezawa S, Yamamoto T, Yamakawa H, Muto C, Hosono M, Hattori K, Higashino K, Yutsudo T, Iwamoto H, Kondo Y, Sakagami M, Togame H, Tanaka Y, Nakano T, Takemoto H, Arisawa M, Shuto S (2012). Conformational restriction approach to beta-secretase (BACE1) inhibitors: effect of a cyclopropane ring to induce an alternative binding mode. J Med Chem.

[100] Yu G (2000). Nicastrin modulates presenilin-mediated notch/glp-1 signal transduction and betaAPP processing. Nature.

[101] Zhou L, Barao S, Laga M, Bockstael K, Borgers M, Gijsen H, Annaert W, Moechars D, Mercken M, Gevaert K, De SB (2012). The neural cell adhesion molecules L1 and CHL1 are cleaved by BACE1 protease in vivo. J Biol Chem.

[102] Zhu Z (2010). Discovery of cyclic acylguanidines as highly potent and selective beta-site amyloid cleaving enzyme (BACE) inhibitors: Part I--inhibitor design and validation. J Med Chem.

